# Biochemistry of Autoinflammatory Diseases: Catalyzing Monogenic Disease

**DOI:** 10.3389/fimmu.2019.00101

**Published:** 2019-01-31

**Authors:** David B. Beck, Ivona Aksentijevich

**Affiliations:** Metabolic, Cardiovascular and Inflammatory Disease Genomics Branch, National Human Genome Research Institute, National Institutes of Health, Bethesda, MD, United States

**Keywords:** autoinflammation, innate immunity, mutations, enzyme deficiency, metabolic sensors, ubiquitination, protein homeostasis

## Abstract

Monogenic autoinflammatory disorders are a group of conditions defined by systemic or localized inflammation without identifiable causes, such as infection. In contrast to classical primary immunodeficiencies that manifest with impaired immune responses, these disorders are due to defects in genes that regulate innate immunity leading to constitutive activation of pro-inflammatory signaling. Through studying patients with rare autoinflammatory conditions, novel mechanisms of inflammation have been identified that bare on our understanding not only of basic signaling in inflammatory cells, but also of the pathogenesis of more common inflammatory diseases and have guided treatment modalities. Autoinflammation has further been implicated as an important component of cardiovascular, neurodegenerative, and metabolic syndromes. In this review, we will focus on a subset of inherited enzymatic deficiencies that lead to constitutive inflammation, and how these rare diseases have provided insights into diverse areas of cell biology not restricted to immune cells. In this way, Mendelian disorders of the innate immune system, and in particular loss of catalytic activity of enzymes in distinct pathways, have expanded our understanding of the interplay between many seemingly disparate cellular processes. We also explore the overlap between autoinflammation, autoimmunity, and immunodeficiency, which has been increasingly recognized in patients with dysregulated immune responses.

## Introduction

Autoinflammatory disorders are characterized by recurrent or persistent systemic or organ specific inflammation without inciting event and classically present with elevated acute phase reactants (1, 2). Characterization of these disorders has primarily focused on familial forms of disease due to highly penetrant mutations as was the case with the identification of the *MEFV* gene responsible for Familial Mediterranean Fever (FMF) and mutations in the *TNFRSF1A* gene as the cause of dominantly inherited TRAPS (Tumor Necrosis Factor Receptor Associated Periodic Syndrome). As genetic sequencing technology and analysis have improved and cost has decreased, there have now been nearly 30 genes identified as causative for autoinflammatory disorders (3). Many of the earliest identified monogenic autoinflammatory diseases were directly related to constitutive inflammasome activation and include FMF and cryopyrinopathies, or loss of a critical inhibitory mechanism as in deficiency of IL-1 (DIRA) or IL-36 (DITRA) receptor antagonist leading to imbalanced cytokine receptor signaling (4–9). Examples such as these have led to classification systems focused on the primary molecular pathways that are altered and thus diseases have been denoted as inflammasomopathies, interferonopathies, and NF-kB related autoinflammatory disorders (10–13). These classifications have helped identify shared mechanisms of disease pathogenesis and principles of treatment. Generally, autoinflammatory disorders are due to gene dysregulation restricted to hematopoietic lineages, whereas involvement of non-inflammatory cells is limited. Although most monogenic autoinflammatory disorders can be placed into this paradigm, many newly identified disorders seem to defy this classification and they have revealed a role for pathways not previously linked to immune function.

Here we will focus on classifying a subset of disorders by the specific biochemical deficiency as opposed to the clinical manifestations or immune mechanism that is disrupted (Table 1). These disorders will be organized by the affected cellular function to highlight the unexpected links between specific biochemical processes and immune dysregulation. We will review disorders that are due to loss of a enzymatic activity and how these diseases may reveal important aspects not only of immunology but of basic cellular signaling. Enzymatic deficiencies offer unique potential treatment strategies based on either accumulation of toxic substrates or loss of catalytic products and can theoretically be treated with enzyme replacement therapy.

**Table 1:** Summary of diseases, genes, and inheritance for autoinflammatory disorders discussed.

**Disease**	**Acronym**	**MIM disease**	**Inheritance**	**Gene/protein**	**Transcript ID**
Sideroblastic anemia, B-cell immunodeficiency, developmental delay and periodic fevers	SIFD	616084	AR	*TRNT1*/TRNT1	NM_001302946
Majeed syndrome	–	609628	AR	*LPIN2*/LPIN2	NM_014646.2
PLCγ2-associated antibody deficiency, and immune dysregulation	PLAID	614468	AD	*PLCG2/*PLCγ2	NM_002661.3
Autoinflammation, PLCγ2-associated antibody deficiency, and immune dysregulation	APLAID	614878	AD	*PLCG2*/PLCγ2	NM_002661.3
Monogenic systemic JIA/IBD	–	–	AR	*C13ORF31/*LACC1/FAMIN	NM_153218
Mevalonate kinase deficiency	MKD/HIDS	251170/260920	AR	*MVK*/MVK	NM_000431.3
Haploinsufficiency A20	HA20	616744	AD/*De novo*	*TNFAIP3*/A20	NM_006290.3
Otulipenia/Otulin-related autoinflammtory syndrome	ORAS	615712	AR	*OTULIN*/OTULIN	NM_138348.5
RIPK1 deficiency	–	618108	AR	*RIPK1*/RIPK1	NM_003804
Deficiency of adenosine deaminase 2	DADA2	615688	AR	*CECR1*/*ADA2*/ADA2	NM_001282225.1

## Disorders Due to Disruption of Protein Translation and Homeostasis

### tRNA Nucleotidyltransferase, CCA-Adding, 1 (TRNT1) Deficiency

The *TRNT1* gene encodes a ubiquitously expressed tRNA nucleotidyltransferase, CCA-Adding, 1 (TRNT1) that is essential for protein synthesis. TRNT1 adds and repairs the conserved CCA sequence at the 3′ end of all precursor cytosolic and mitochondrial transfer ribonucleic acids (tRNAs), a step necessary for the attachment of conjugate amino acids (14). TRNT1 also regulates RNA stability through tRNA decay mechanisms and may play an important role in reducing levels of non-coding RNAs (15). TRNT1 is localized to the mitochondria via a 41 amino acid transit peptide and is expressed in all tissues. The crystal structure of human TRNT1 (PDB ID:1Ou5) shows that the protein functions as a homodimer via intermolecular disulfide bond (16). Complete deficiency of *Trnt1* in mice is embryonic lethal further highlighting the essential function of this gene.

**Figure 1:**
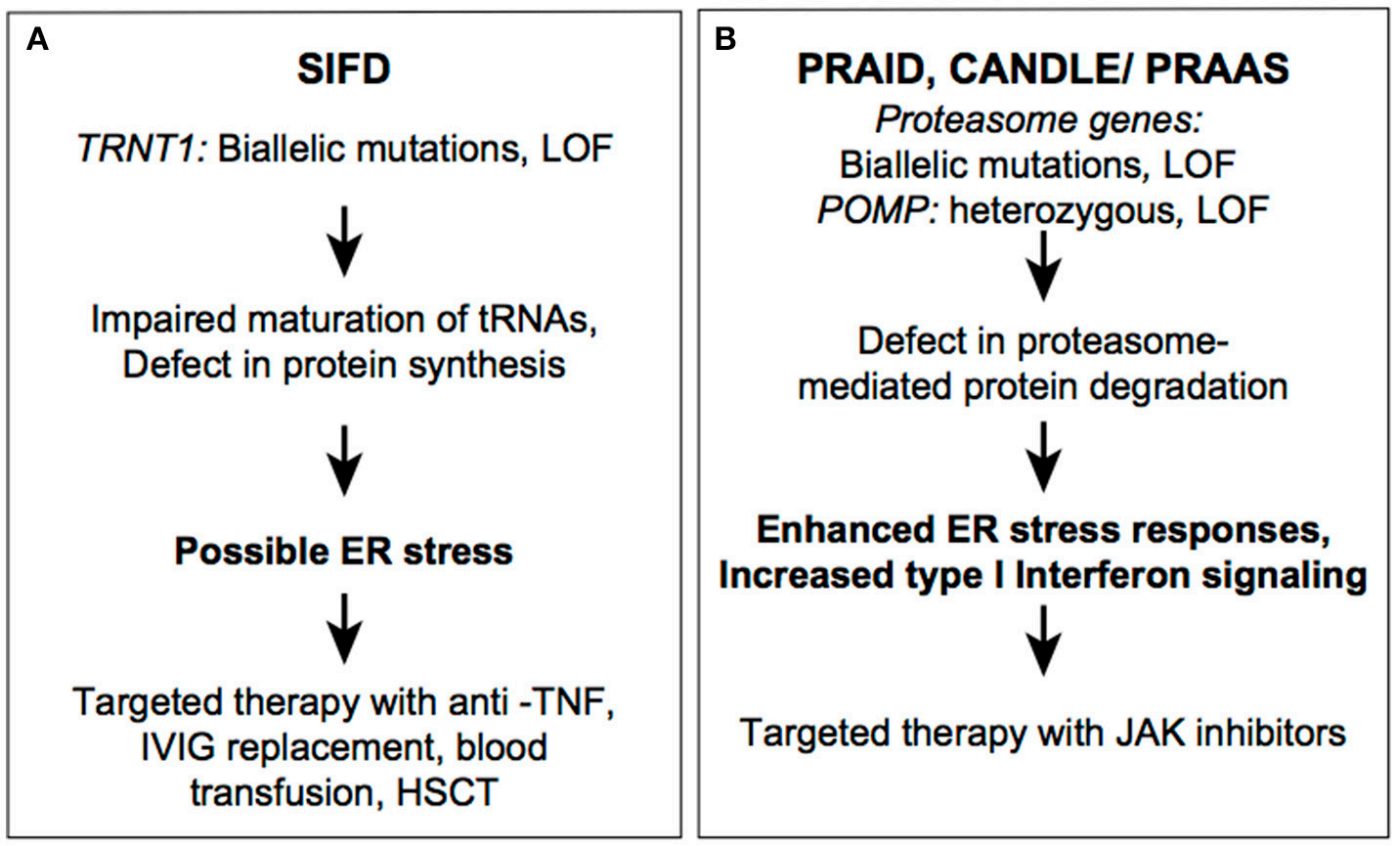
Autoinflammatory diseases due to dysregulation in proteostasis. SIFD **(A)** and CANDLE **(B)** syndromes.

Bi-allelic loss of function mutations in *TRNT1* lead to a recessively inherited syndrome named SIFD for sideroblastic anemia, B-cell immunodeficiency, developmental delay, and periodic fevers (Figure 1A) (17, 18). Given the ubiquitous expression of TRNT1, it is not surprising that reduced function of the enzyme leads to a complex phenotype. To date, more than 30 patients have been reported with significant clinical and immunologic heterogeneity (17, 19–22). At the severe end of the spectrum are patients with neonatal-onset severe anemia and prominent extramedullary erythropoiesis, profound immunodeficiency, metabolic and neurological abnormalities (17). In this first published cohort of 12 patients, median survival was 48 months and seven patients died due to cardiac or multiorgan failure. Recurrent fever has been reported in most but not all patients with SIFD. Immunodeficiency in SIFD is primarily due to defects in B cells differentiation and can manifest early in life or can be progressive and present later (23). T and NK cell numbers are in the low-normal range and some patients also carry a diagnosis of combined variable immunodeficiency but without serious bacterial or viral infections. At the milder end of the spectrum are patients with non-syndromic retinitis pigmentosa and subtle hematological features (24, 25).

SIFD-associated variants are loss-of-function and they include missense, non-sense, frameshift, and splice site mutations. *TRNT1* pathogenic variants are either novel or have a low frequency in the general population. As is the case with many other recessively inherited diseases, a subset of patients were identified in founder populations with homozygous pathogenic variants. Hypomorphic mutations in TRNT1 reduce protein expression, affect protein stability, or alter its catalytic efficiency (18, 26). So far, no patients have been identified with biallelic non-sense or frameshift mutation, further emphasizing that residual TRNT1 protein is essential for development. In TRNT1-deficient cells, maturation of nuclear and mitochondrial tRNAs is impaired, which leads to a defect in global protein synthesis (21, 27). As result, these cells are unable to maintain protein homeostasis under stress conditions. Protein degradation pathways, which are essential for clearance of unprocessed/misfolded proteins, eventually become insufficient to remove an excessive load of misfolded proteins in mutant cells. Accumulation of misfolded proteins can then result in cell death and release of various proinflammatory cytokines through the unfolded protein response (UPR). Although ER stress and activation of the UPR has not been experimentally demonstrated in TRNT1-deficient cells, there are examples of other similar systemic inflammatory diseases of dysregulated protein homeostasis, such as Chronic atypical neutrophilic dermatosis with lipodystrophy and elevated temperature (CANDLE) and POMP-related autoinflammation and immune dysregulation disease (PRAID). Both CANDLE and PRAID syndrome result from defects in proteasome assembly leading to the accumulation of ubiquinated proteins triggering cellular stress and the type 1 interferon (IFN) response (Figure 1B) (28, 29). In addition to the UPR stress-mediated inflammatory response, reactive oxygen species (ROS) accumulation has been observed in cultured SIFD patients' fibroblasts, and elevated ROS levels can activate other inflammatory pathways such as the NLRP3 inflammasome and type I interferon response (21, 30). Furthermore, some clinical manifestations of SIFD resemble patients with mitochondrial disorders and patient-derived fibroblasts showed decrease in cellular respiration and oxidative phosphorylation (31).

Thus, far, it has not been investigated why hypomorphic mutations in *TRNT1* cause severe defects in differentiation of hematopoietic cells, but it may have to do with tissue-specific regulatory functions or the specific metabolic demands of immune cells. Similar tissue specific clinical manifestations have been identified in genetic syndromes with loss of tRNA synthetases, enzymes responsible for tRNA conjugation to specific amino acids, despite their essential function across all tissues (32).

Most patients are treated symptomatically with blood transfusions, IgG replacement therapy, and corticosteroids, while the disease morbidity and mortality remains high. Necessity for blood transfusions for anemia is often increased during fevers. Anti-TNF therapy suppresses inflammation reducing the need for blood transfusions, and improving growth, although it is not clear if non-immune functions improve under these therapies (21). Molecular diagnosis in early life is crucial to prevent some of the severe disease consequences. Hematopoietic stem cell transplantation (HSCT) has been attempted and has helped with hematological features, however one patient died of transplant-related complications (17).

## Second Messenger Mediated Diseases

### LPIN2-Deficiency

LPIN2 is a member of the lipin family of enzyme, which function in glycerolipid biosynthesis (33, 34). There are 2 other lipin family member enzymes, LPIN1, and LPIN3. LPIN2 is a phosphatidate phosphatase (PAP) that catalyzes the conversion of phosphatidic acid to diacylglycerol (DAG), a critical byproduct necessary for the production of triacylgycerol, phophatidylcholine, and phosphatidylethanolamine (35). DAG works as a secondary lipid messenger by activating protein kinases and RAS signaling pathways among others (36). In addition to the PAP activity, LPIN1 and 2 may function as a transcriptional co-activator with peroxisome proliferator-activated receptors (PPARα) (37). LPIN1 and LPIN2 are expressed in macrophages and seem to have opposite roles in regulating immune responses (38, 39). While LPIN1 acts as a proinflammatory mediator during Toll-like receptor (TLR) signaling, LPIN2 downregulates proinflammatory signaling induced by saturated fatty acids (40). *In vitro* depletion of *LPIN2* causes increased expression of IL-6 and TNF in macrophages. *In vivo* studies of LPIN2 function have also demonstrated its role as a negative regulator of the NLRP3 inflammasome and TLR4 signaling (41). LPIN2-deficient mouse bone marrow derived macrophages (BMDM) display low cholesterol levels, an increase in ATP-promoted potassium flux, and increased activity of the PTX_7_ receptor. Thus, reduced function of LPIN2 results in upregulation of the inflammatory pathways and leads to overproduction of IL-1β, IL-18, and TNF cytokines. Similar results were obtained in primary patient-derived macrophages. These studies established a critical link between lipid biosynthesis and inflammation.

**Figure 2:**
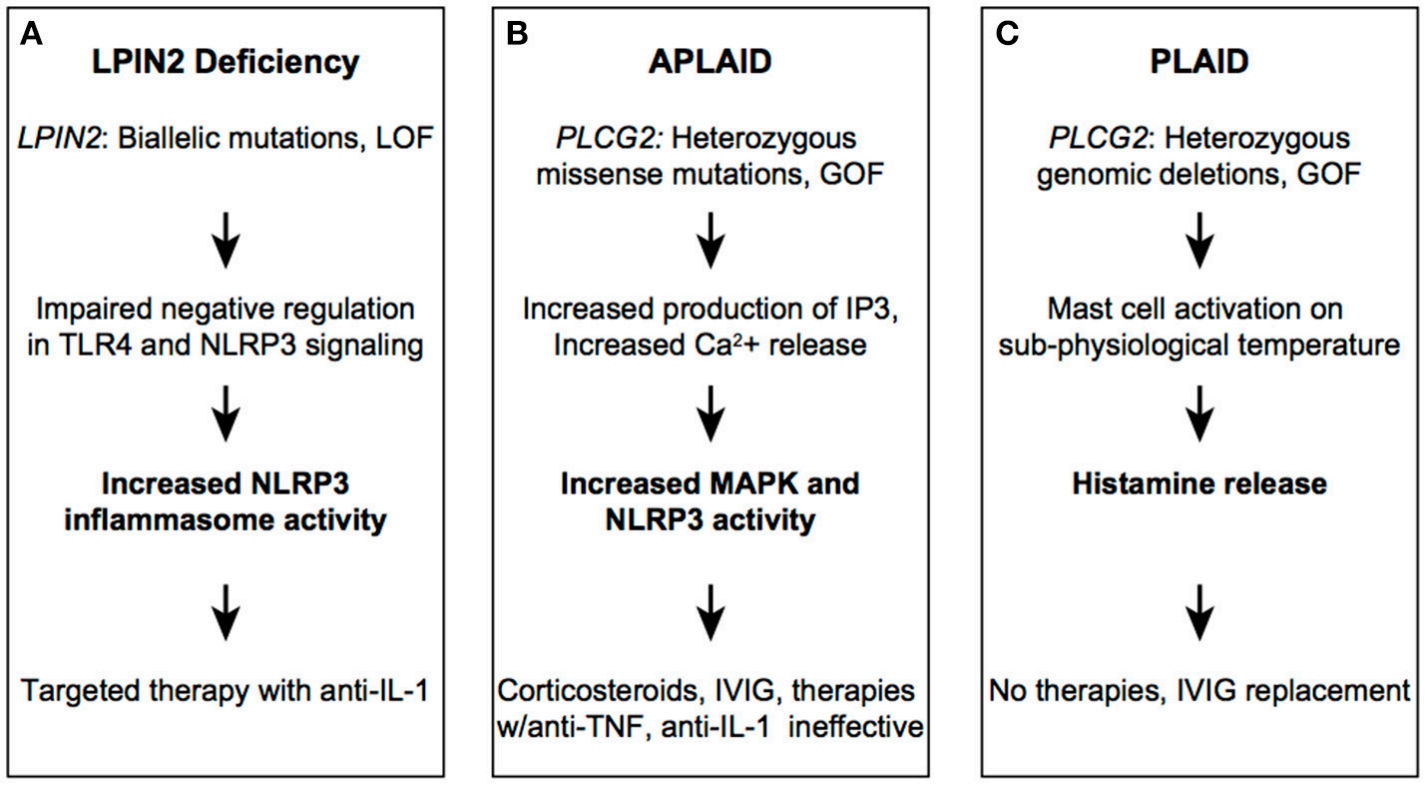
Autoinflammatory diseases due to dysregulation in lipid-mediated signaling. LPIN2 **(A)**, PLAID **(B)**, and APLIAD **(C)** syndromes.

*LPIN2* loss of function in humans leads to Majeed Syndrome, a rare, recessively inherited disorder that is characterized by the triad of early-onset chronic recurrent multifocal osteomyelitis (CRMO), dyserythropoietic anemia (typically microcytic), and neutrophilic skin lesions (Figure 2A) (42, 43). While CRMO and skin inflammation can be explained by a loss of LPIN2 anti-inflammatory function, the molecular mechanism of anemia is less clear. Interestingly, LPIN1 is highly expressed in muscle, and loss of enzymatic activity leads to a recessively inherited muscle disease (44). Given the ubiquitous expression of *LPIN1* and *LPIN2* and the widespread importance of glycerolipids in diverse cellular functions, it is intriguing why deficiency of these enzymes would cause these very distinct, tissue-specific phenotypes. To date, 14 cases of Majeed syndrome have been reported, all in consanguineous families of Middle Eastern ancestry. As all patients carry a private homozygous mutation there is a limited number of identified pathogenic variants (*Infevers*). Causal variants include mostly non-sense and splice site mutations that lead to reduced protein expression. A single pathogenic missense variant, p.Ser734Leu, was shown *in vitro* to abolish the PAP activity of LPIN2, suggesting that this activity is important for the pathogenesis of the disease (45).

IL-1 blockade results in dramatic improvement in clinical and laboratory parameters of inflammation in Majeed syndrome, confirming that loss of LPIN2 in these patients leads to autoinflammation via enhanced IL-1β production (46).

### PLCG2-Associated Diseases

Phospholipase C (PLC) enzymes have a key role in the regulation of variety of cellular functions (47). The PLC family of enzymes include PLCγ1 and PLCγ2, encoded by *PLCG1* and *PLCG2* genes, respectively. These enzymes catalyze the hydrolysis of phospholipids and generate secondary messengers that provide a signal between many cellular receptors with downstream intracellular pathways. While *PLCG1* is ubiquitously expressed, *PLCG2* is highly expressed in hematopoietic cells. PLCγ2 is activated by immune receptors such as B cell and Fc receptors and has an important role in mediating innate immune responses (48). In response to receptor stimulation, PLCγ2 catalyzes the formation of the second messengers inositol triphosphate (IP_3_) and DAG from phosphatidylinositol 4,5-bisphosphate (PIP2). IP_3_ binds to IP_3_ receptors on the endoplasmic reticulum, resulting in calcium release and subsequent activation of various signaling pathways including mitogen activated protein kinases (MAPK). DAG remains bound to the cell membrane where it can activate protein kinase C (PKC) and other kinases. Pathogenic mutations in *PLCG2* lead to two distinct phenotypes: PLCγ2-associated antibody deficiency and immune dysregulation (PLAID) and autoinflammation, antibody deficiency, and immune dysregulation (APLAID) (Figures 2B,C).

PLAID is a dominantly inherited disease characterized by cold-induced urticarial or blistering rash with onset in infancy, variable degrees of immunodeficiency and autoimmunity, and skin granuloma formation (49). In some patients, the skin lesions get worse over time leading to tissue destruction. APLAID is also dominantly inherited and presents early in life with fever, blistering, or erythematous relapsing skin lesions often triggered by heat and sweating, arthralgia, ocular inflammation, enterocolitis, and progressive interstitial lung disease (50). To date over thirty patients with PLAID and only two patients with APLAID have been reported (51). The main distinguishing features between PLAID and APLAID are that PLAID patients have cold-induced urticaria and prominent autoimmune features as compared to APLAID patients. Features of autoimmunity were found in about 25% of patients with PLAID. B cell immunodeficiency is in the spectrum of both diseases, and manifests with recurrent bacterial respiratory and GI infections. Common laboratory findings include antibody deficiency (low serum IgG and IgM) and decreased levels of circulating CD19+ and class-switched memory B cells.

The disease pathophysiology is due to a complex combination of temperature-sensitive, cell-specific gain and loss-of-function variants in the PLCγ2 signaling pathway (52). PLAID families were found to have in-frame genomic deletions spanning the cSH2 autoinhibitory domain of PLCγ2. This domain blocks the active site of the enzyme and deletion of this region results in diminished PLCγ2-mediated signaling at physiologic temperature but enhanced signaling at sub-physiologic temperatures. Primary B and NK cells from PLAID patients have an anergic phenotype while mast cells spontaneously activate when exposed to lower temperature due to loss of the autoinhibitory domain. On the other hand, APLAID patients, who carry a gain-of-function missense mutation instead of a deletion in the same cSH2 autoinhibitory domain of PLCγ2, have very different cellular and clinical phenotypes. The gain-of-function mutation identified in APLAID patients, p.Ser707Tyr, causes temperature-independent production of IP_3_, intracellular Ca^2+^ release and ERK phosphorylation in patient-derived PBMCs (50). In addition, there is evidence for Ca-dependent activation of the NLRP3 inflammasome in primary cells (53). Collectively, these data suggest constitutive hyper activation of myeloid cells in APLAID.

PLAID and APLAID patients with noticeable immunodeficiency require immunoglobulin replacement therapy, while patients with a severe inflammatory phenotype have proven difficult to treat. Although the NLRP3 inflammasome was shown to have a role in mediating inflammation in APLAID, IL-1 inhibitors have been mostly ineffective. Similarly, other cytokine inhibitors have been trialed but were ineffective in suppressing disease activity. This observation suggests function for other yet unknown pathways in the disease pathogenesis.

## Metabolic Sensors Implicated in Autoinflammatory Diseases

### LACC1 Deficiency

*LACC1* encodes for the protein FAMIN (Fatty Acid Metabolism and Immunity Nexus), which has homology to bacterial multicopper oxidoreductase enzymes (54, 55). FAMIN interacts with fatty acid synthase (FASN) and localizes to the peroxisome. FAMIN is predominantly expressed in macrophages, where it regulates fatty acid oxidation and lipogenesis to maximize metabolism (55). The exact mechanism of how FAMIN controls lipid balance is not clear but patient monocytes with different *LACC1* alleles have altered laccase (phenol-oxidoreductase) suggesting loss of this enzymatic activity may be the mechanism of disease (56, 57).

**Figure 3:**
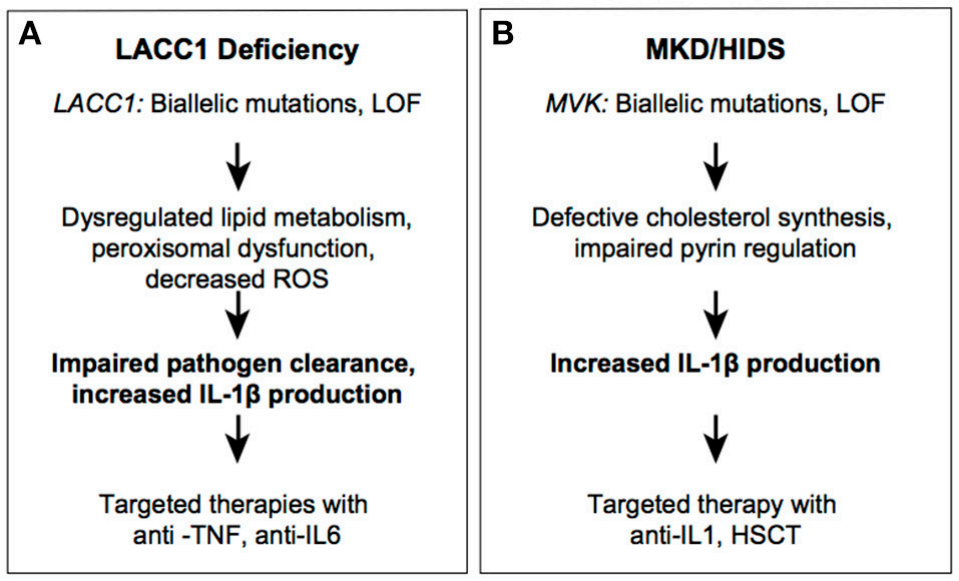
Autoinflammatory diseases due to impaired metabolic sensors. LACC1 **(A)** and MVK **(B)** syndromes.

The first link between *LACC1* and disease was identified through an association with leprosy and Crohn's disease (CD) by genome wide association studies (GWAS) (58–61). Interestingly leprosy and Crohn's disease are both granulomatous disorders, and share the same *LACC1* risk allele, the specific missense p.Ile254Val variant (rs3764147; MAF = 0.27). Later studies have demonstrated an association between a SNP (rs2121033; MAF = 0.27) downstream of *LACC1* with Behcet's Disease (Figure 3A) (62, 63). It is possible that these two susceptibility alleles are in linkage disequilibrium and therefore inherited on the same haplotype. *LACC1* was subsequently identified as a cause of recessively inherited monogenic CD, based upon a single consanguineous Saudi Arabian family with multiple members presenting with early-onset disease (64). The novel p.Cys284Arg variant segregated with the disease phenotype in this large family with four affected and seven unaffected members. This mutation is predicted to be detrimental for protein stability and activity, although this has not been confirmed experimentally. *LACC1* was further implicated in monogenic autoinflammatory disease through identification of families with recessively inherited systemic juvenile idiopathic arthritis (SoJIA) (65). The same mutation, p.Cys284Arg, identified in the family with CD, was identified in 13 children affected with juvenile idiopathic arthritis from five consanguineous Saudi Arabian families. All patients had symmetrical polyarthritis, the characteristic quotidian fevers, evanescent rash and were homozygous for the missense mutation p.Cys284Arg (65). Interestingly, none of the patients with inflammatory arthritis had CD or ulcerative colitis. Thus, the same population-specific variant has been linked to distinct inflammatory phenotypes. Two recent studies reported 19 additional patients with primarily juvenile idiopathic arthritis, all from Middle Eastern consanguineous families, with distinct genotypes including p.M1I, p.R414^*^, p.I330del, p.I254V, p.Cy370Tyrfs^*^6, and p. T276fs^*^2 in *LACC1* (66, 67). Some of these mutations have been shown to result in decreased protein expression, confirming that the disease is caused by loss of function of FAMIN.

Effective treatment modalities reported include TNF blockade with adalimumab or IL-6 inhibition with tocilizumab (66). Given the fact that *LACC1/*FAMIN controls reactive oxygen species production in macrophages, loss of this enzyme was predicted to result in decreased IL-1β production. However, knockout and knock-in disease mutation murine models show increased IL-1β, likely due to compromised pathogen clearance (55). Similar findings were made in patient-derived cells with a p.I254V homozygous variant, which revealed decreased ROS production and decreased NOD2-dependent bacterial clearance (56). The central function of *LACC1* in granulomatous disease and pathogen clearance is similar to that of *NOD2*, a key pathogen recognition receptor mutated in a Mendelian granulomatous disease, Blau Syndrome, and polygenic CD. Because the same *LACC1* mutations, p.Ile254Val and p.Cys284Arg, have been associated with multiple phenotypes including, leprosy, BD, SoJIA and early-onset CD, it is likely that environmental factors, such as microbiota, play an important role in disease expressivity and manifestations. The study of phenotypes associated with mutations in *NOD2* and *LACC1* implies that environmental factors may be important in the pathogenesis of more common granulomatous diseases including sarcoidosis (68). The critical position of FAMIN in cellular energy homeostasis reveals unique regulatory functions that exist in phagocytes to ensure a balance of energy sources available for clearance of pathogens.

### Mevalonate Kinase-Associated Diseases

Another example of dysregulated immunometabolism is mevalonate kinase deficiency/hyper IgD syndrome (MKD/HIDS). Mevalonate kinase (MVK) is a key enzyme in the biosynthesis of cholesterol and isoprenoids, catalyzing the conversion of mevalonic acid to mevalonate-5-phosphate. Through this role in cholesterol synthesis, MVK regulates levels of geranylgeranyl pyrophosphate, which is important for prenylation and regulation of the small GTPases. RhoGTPases inhibit the pyrin inflammasome by activating protein kinase N enzymes (PKNs), which suppress pyrin function (9). Protein geranylgeranylation is also critical for TLR-induced activation of phosphatidylinositol-3-OH kinase (PI(3)K) through the interaction between the small GTPase KRAS and the PI(3)K catalytic subunit p110delta. Compromised (PI(3)K) activity results in constitutive activation of pyrin (69). Thus, loss of MVK activity leads to reduced prenylation of GTPases, which in turn results in increased activity of the pyrin inflammasome and excessive production of IL-1β.

Recessive loss of function mutations in *MVK* can result in a spectrum of disease ranging from severe multisystem disease (mevalonic aciduria; MKD) to a milder autoinflammatory disease (HIDS) (Figure 3B) (70). Patients with MKD manifest dysmorphic features, psychomotor retardation, progressive cerebellar ataxia, and systemic inflammation. Patients with HIDS have episodes of high fever, rash, abdominal pain, aphthous ulcers, pharyngitis, swollen lymph nodes, and arthralgia/arthritis. These symptoms are often triggered by immunization or other stresses. Cells from patients with HIDS still have residual MVK enzymatic activity (about 1–10% of the activity found in healthy control cells), whereas patients with MKD have complete deficiency in enzyme activity (71, 72). Most severe MKD-associated mutations create truncated proteins, whereas HIDS-associated mutations are missense substitutions and are thought to impair MVK stability (73, 74). The inflammatory phenotype in patients with HIDS is ameliorated with anti-IL-1 therapy consistent with the finding that MVK-deficient PBMCs secrete higher levels of IL-1β (75–77).

## Post-translation Modifying Enzymes in Autoinflammatory Diseases

Several recently described autoinflammatory disorders highlight the role of post-translational modifications (PTMs), in the regulation of TNF and IL-1β signaling pathways and NF-κB activation (10, 78). Ubiquitination is the covalent attachment of an evolutionarily conserved 76-amino acid ubiquitin (Ub) protein to target substrates in the form of a monomer or polymers (ubiquitin chains; Ub chains). Ubiquitin chains can be conjugated at different lysine residues (K6, K11, K29, K33, K48, K63) along with the amino terminal methionine (M1), which can determine the fate of the modified protein. Proteins conjugated with Lys48 (K48) Ub chains are targeted for degradation via the ubiquitin-proteasome system (UPS), while Lys63 (K63) linked and linear Ub chains have essential roles in promoting signaling cascades. Ubiquitination can be reversed by a class of enzymes known as deubiqutylases or deubiquitinases (DUBs). There are more than 100 known DUBs expressed in various cells and with different degrees of specificity for Ub chains. Several DUBs are highly expressed in hematopoietic cells where they function as negative regulators of NF-kB signaling (A20, OTULIN, CYLD, and Cezanne). Mutations in genes encoding some of these DUB enzymes, along with other PTM enzymes such as kinases, have been recently implicated in autoinflammatory disorders.

### Haploinsufficiency of *TNFAIP3/*A20 (HA20)

TNFAIP3/A20 has two enzymatic activities that synergize to restrict inflammatory responses: it has deubiquitinase activity by which it causes hydrolysis of K63 Ub linkages in receptor signaling complexes, and it has E3 Ub ligase activity through which substrates are modified with K48 Ub chains to target them for proteasomal degradation (79). TNFAIP3/A20 is a 790-residue protein that consists of an amino-terminal ovarian tumor domain (OTU) followed by 7 zinc finger domains (ZFs). The ZnF4 domain is essential for A20 E3 ligase activity and dimerization. The protein has a critical role as negative regulator of canonical NF-κB signaling. A20 null mice exhibit multi-organ inflammation, cachexia, and early lethality, while conditional A20 knockout in B cells, T cells and/or epithelial cells alone do not lead to spontaneous inflammation suggesting that the inflammatory phenotype is specific to myeloid cells (80).

**Figure 4:**
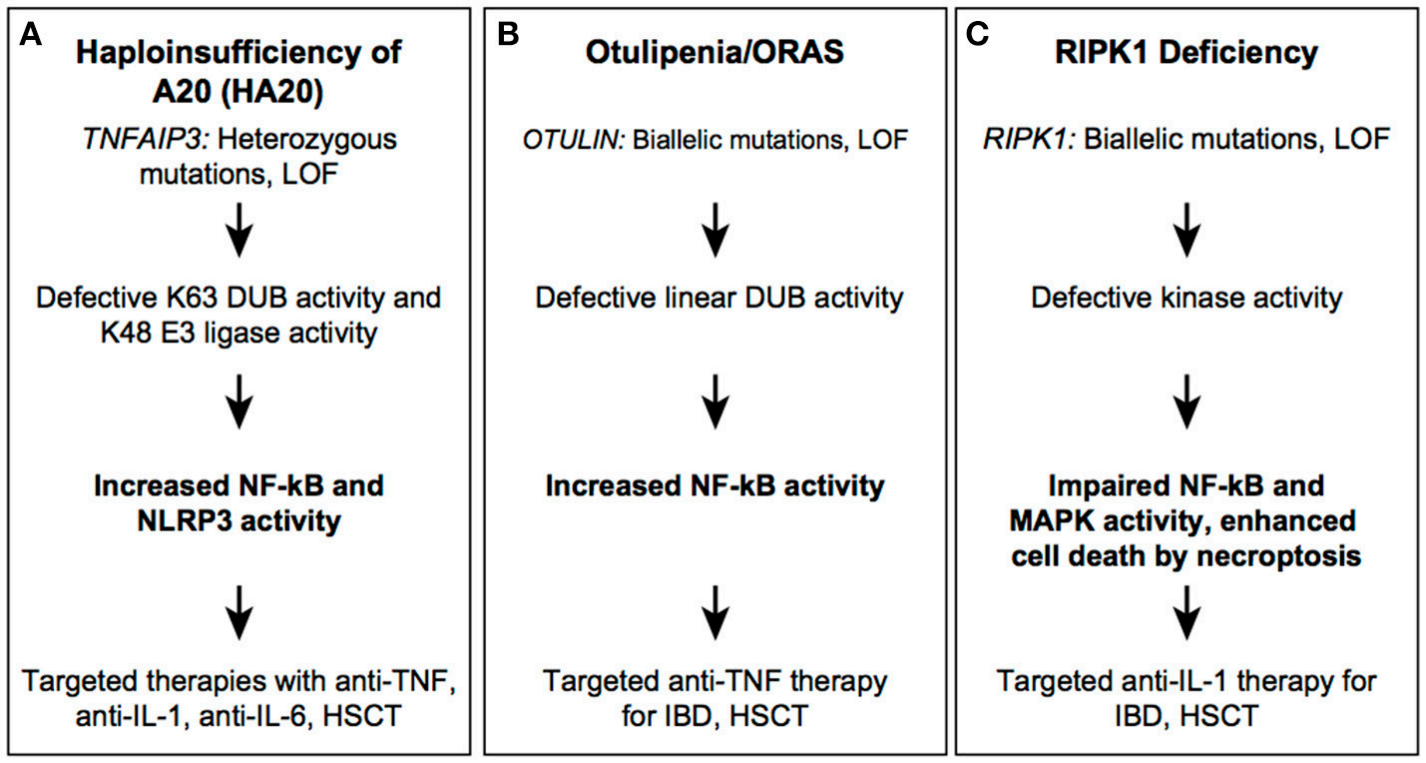
Autoinflammatory diseases due to dysregulation in post-translational modification. HA20 **(A)**, Otulipenia **(B)**, and RIPK1 deficiency **(C)** syndromes.

Heterozygous loss-of-function mutations in TNFAIP3/A20 are associated with an autoinflammatory disease named haploinsufficiency of A20 (HA20) (Figure 4A) (81). Most patients with HA20 present with an early-onset autoimmune/autoinflammatory phenotype, with a variety of organ-specific features that are analogous to diseases such as Behcet's disease, systemic lupus erythematosus, Hashimoto's thyroiditis, autoimmune lymphoproliferative syndrome, and Crohn's disease. Many patients present with oral and genital ulcers, which are uncommon symptoms in other monogenic autoinflammatory diseases (82–86). There is substantial variability in expressivity of disease even among patients with the same genotype. Recently, large deletions on chromosome 6 encompassing up to 55 genes including *TNFAIP3*, were identified in patients with systemic inflammation, psychomotor and growth delay, and *situs inversus* (87, 88). The heterotaxy (abnormal organ arrangement) was likely the consequence of the deleted *CITED2* gene (89, 90). Thus, anti-inflammatory therapy should be considered in patients with a complex phenotype and heterozygous genomic deletions on Chr. 6q23-q24.

HA20-associated mutations are mostly located within the OTU domain and lead to truncated proteins of different length, with some partial protein products stably expressed in patient cells (91). Whether these truncated protein affect signaling remains unclear, although a dominant negative mode of action for these byproducts could mechanistically explain the phenotype. In addition to germline mutations in *TNFAIP3*, a low-frequency somatic mutation was reported in two unrelated Japanese families, which is of particular concern for genetic counseling and disease reoccurrence risk stratification (84). Irrespective of mutation type and position, patients with active disease have high serum levels of many proinflammatory cytokines such as IL-1β, IL-6, IL-9, IL-17, IL-18, TNF, suggesting constitutive activation of canonical NF-κB and NLRP3 inflammasome pathways (81, 84). Therapies with cytokine inhibitors, such as anti-TNF, anti-IL-1, and anti-IL-6 have been successfully employed in controlling disease activity. HSCT was curative in one reported patient (92).

### Otulipenia/Otulin-Related Autoinflammatory Syndrome (ORAS)

OTULIN is a highly conserved deubiquitinase that hydrolyzes Met1-linked (linear) Ub chains from conjugated substrates. OTULIN has a critical role in regulation of angiogenesis and is required for craniofacial and neuronal development by regulating canonical Wnt signaling (93). OTULIN also functions as a negative regulator of the canonical NF-kB pathway by deubiquitinating the linear ubiquitination chain assembly complex (LUBAC) (94). LUBAC, which consists of HOIP, HOIL1, and SHARPIN, catalyzes linear ubiquitination and is essential for NF-κB signaling (95). Conditional knockout of *Otulin* in immune lineages results in a viable but severe inflammatory phenotype in mice with a more severe phenotype in the KO myeloid lineage than lymphoid lineage (96).

Recessively inherited, loss of function mutations in the OTU domain of OTULIN are associated with the early-onset severe inflammatory disease (Figure 4B) (96, 97). Patients present with failure to thrive, recurrent fevers, rash, joint swelling, and gastrointestinal inflammation. The cutaneous manifestations include painful erythematous rashes, subcutaneous skin nodules, and lipodystrophy. In one highly inflamed patient, skin biopsy showed neutrophilic dermatitis and panniculitis. To date, three mutations (2 missense and 1 frameshift) in five individuals from Middle Eastern, consanguineous families, have been reported. The phenotypic characteristics of OTULIN deficiency will expand with increased awareness and identification of new mutations. Pathogenic mutations affect the catalytic activity of OTULIN thereby impairing removal of linear ubiquitination; mutant cells accumulate linear Ub chains on various substrates such as IKKγ/NEMO, RIPK1, and ASC (97). Increased linear ubiquitination of IKKγ and RIPK1 results in constitutive activation of the canonical NF-kB pathway. Patient-derived immune cells produce higher levels of many proinflammatory cytokines that are secreted both by myeloid cells and T cells, such as IL-17 and IFNγ. Therapy with TNF inhibitors has been very effective in suppressing systemic inflammation and in improving growth and development. Hematopoietic stem cell transplantation (HSCT) may also hypothetically rescue the hematological phenotype although it has not been attempted due to the favorable response to treatment with TNF-inhibitors.

### Receptor Interacting Protein Kinase 1 (RIPK1)-Associated Immunodeficiency and Autoinflammation

Receptor Interacting Protein Kinase 1 (RIPK1) is a widely expressed serine/threonine kinase that regulates TNFR (TNF receptor) signaling and cell death pathways (98). RIPK1 plays an important role in the regulation of immune responses through its dual functions either in activating NF-κB signaling or initiating cell death programs. RIPK1 serves as a scaffold in plasma membrane-associated protein complexes that transmit signals from TNFR1, TLR2, and TLR4 cell surface receptors. Stimulation of these receptors activates the canonical NF-κB pathway, MAPK, and pro-survival genes. Cytoplasmic RIPK1 kinase activity is important for initiation of cell death signaling pathways, apoptosis and necroptosis (99, 100). RIPK1 kinase activity acts primarily in initiating necroptosis, a caspase-independent form of cell death, through auto-phosphorylation, which triggers a protein interaction cascade. During necroptosis, the plasma membrane permeabilizes, releasing damage associated molecular patterns (DAMPs) that can in turn lead to a prolonged immune response. RIPK1 has been extensively studied in murine models, with knockout mice displaying postnatal lethality due to systemic inflammation and increased cell death (101, 102). Inhibition of RIPK1 kinase activity using necroptosis inhibitors, necrostatin-1 and its analogs, can ameliorate a variety of mouse models of disease, including septic shock, myocardial infarction, and amyotrophic lateral sclerosis, along with inflammatory phenotypes such as rheumatoid arthritis and ulcerative colitis implicating necroptosis as a major contributor in disease pathogenesis (103).

Recently, four patients with severe immunodeficiency, gut inflammation, and progressive polyarthritis were identified with *RIPK1* homozygous loss of function alleles (Figure 4C) (104). The disease causing variants are small nucleotide deletions in the N-terminal kinase domain that lead to frameshift and loss of a substantial portion of the protein. The autoinflammatory phenotypic features consists of a GI inflammation with variable onset and severity, which suggests that environmental factors may modulate the disease expressivity. The four described patients suffered from recurrent infections, mostly viral but some bacterial and fungal infection. Immunophenotyping revealed lymphopenia, most prominently of CD4+ T cells, with one patient demonstrating low antibody titers and another with low serum immunoglobulins (IgG, IgM, IgA). RIPK1-deficient patient fibroblasts stimulated with TNF exhibit impaired MAPK activation, reduced cytokine production and decreased cell viability consistent with the immunodeficient phenotype. Similar to fibroblasts, LPS-stimulated patient monocytes showed decreased production of proinflammatory cytokines including IL-6, IL-12, and TNF however, they secreted high levels of IL-1β. Cell death in cultured fibroblasts was predominantly mediated by necroptosis as apoptosis inhibitors were unable to reduce cell death. Although the mechanism of inflammation in humans, like mice, is due to increased cell death via necroptosis, the absence of immunodeficiency in *Ripk1* ^−/−^ mice and the viability of null alleles in humans, hints at a unique role for necroptosis specific to human immune cells.

This disease phenotype is fairly severe and with high mortality. As IL-1β likely contributes to both IBD and arthritis in these patients, anti-IL-1 therapy may be considered to suppress the inflammatory component of the disease although none of the reported patients received targeted IL-1 inhibitors. Three of the patients with RIPK1 deficiency underwent HSCT, two died shortly after transplant due to infectious causes.

## Nucleic Acid Regulation in Autoinflammatory Disease

### Adenosine Deaminase 2 Gene (*ADA2)* Deficiency

Adenosine deaminase (ADA) is an aminohydrolase that regulates purine metabolism and adenosine homeostasis by catalyzing the deamination of adenosine (Ado) and 2′-deoxyadenosine (dAdo) into inosine and deoxyinosine, respectively. Extracellular levels of adenosine increase during hypoxia and tissue injury and can lead to persistent inflammation through activation of adenosine receptors (AdoR) (105). In humans, there are two isoenzymes encoded by different genes, *ADA1* (also known as *ADO*) and *ADA2* (also known as *CECR1*) that have different enzymatic properties. ADA2 has a 100-fold higher Michaelis-Menton constant for adenosine (K_m_ = 2 mM) than ADA1 and is not essential for intracellular deaminase activity. ADA2 is a secreted protein and under physiological conditions is present in low levels in plasma. In addition, ADA2 shares homology with adenosine deaminase growth factors (ADGFs) that have been shown to play a role in development (106). ADA1 and 2 are both highly expressed in immune cells and are critical for the development of the immune system. ADA1 is predominantly expressed in lymphocytes, while ADA2 is secreted by activated myeloid cells (107).

**Figure 5:**
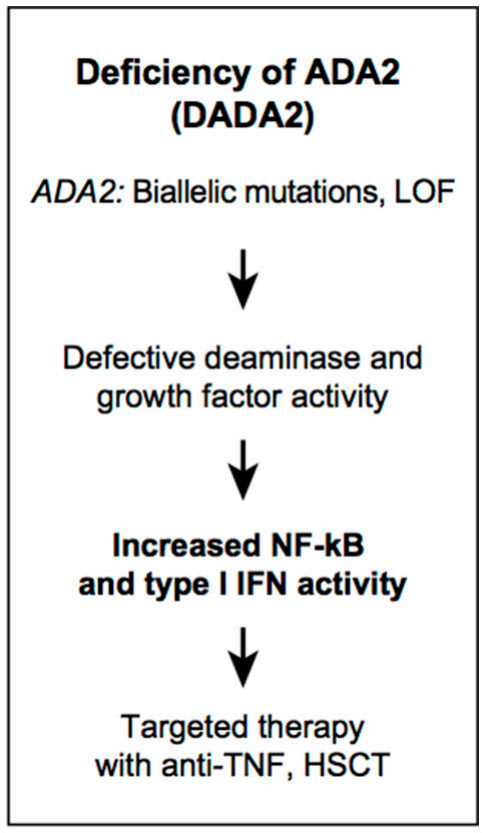
Autoinflammatory diseases due to dysregulation in purine metabolism. DADA2.

The first human disease linked to a defect in ADA1 function was severe combined immunodeficiency. A complete deficiency of ADA1 is fatal early in life, while patients with partial ADA1 deficiency present with milder clinical symptoms, later in childhood. In the absence of ADA1, deoxyadenosine nucleotides accumulate in lymphocytes and are toxic resulting in T-B-NK- SCID (108). Deficiency of adenosine deaminase type 2 (DADA2) is a recessively inherited disease manifesting with fevers, vasculitis, livedo racemosa, early-onset ischemic stroke, liver disease, and mild immunodeficiency (Figure 5) (109, 110). The vasculopathy/vasculitis associated with DADA2 predominantly affects small- and medium-sized arteries with histologic findings consistent with necrotizing ANCA-negative vasculitis or polyarteritis nodosa (109, 111). Livedo racemosa is the most common skin manifestation, and it can be complicated by skin ulcerations, distal arterial occlusion, and sometimes digital necrosis (112). Neurological manifestations result from lacunar ischemic infarcts in the deep-brain nuclei, midbrain, and/or brainstem. Some patients were reported with intracerebral hemorrhage as the first disease manifestation (111, 113). Hematological manifestations were not appreciated initially, however it has become evident that some patients present with hypocellular bone marrow (114–116). Their clinical presentations include aplastic anemia, pure red cell aplasia, lymphopenia, neutropenia, thrombocytopenia, and hypogammaglobilinemia, in particular low IgG and IgM levels. Immunodeficiency is generally milder than in patients with ADA1 deficiency, and resembles common variable immunodeficiency (115). Lymphoproliferative features are also in the spectrum of DADA2 hematopoietic manifestations (117, 118). Autoimmune findings in DADA2 have been found in a small subset of patients, and manifest as autoimmune cytopenias, transiently positive lupus anticoagulant, and systemic lupus. This is consistent with increased type 1 interferon (IFN) gene expression signature in blood samples of patients with DADA2 (119, 120).

To date, there are more than 170 patients diagnosed with DADA2 described from all over the world (121–123). Many DADA2-associated mutations are present at a low frequency in the general population and it is possible that the disease is still underdiagnosed. In addition to standard genetic testing by sequencing, DADA2 can be diagnosed by measuring plasma or serum ADA2 activity. ADA2 activity testing is recommended to confirm the pathogenicity of novel variants identified by sequencing. The molecular mechanisms by which mutations in ADA2 lead to disease are still largely unknown. Proinflammatory cytokines have been identified in skin biopsies and blood samples of DADA2 patients however, the function of ADA2 in differentiation of hematopoietic and endothelial cells has not been comprehensively investigated. Deficiency of ADA2 is associated with monocyte-macrophage polarization toward the M1 subset, and M1 macrophages are known to promote inflammation, although it is not clear if this is directly dependent on adenosine, aminohydrolase catalytic activity on a novel substrate, growth factor function, or another as yet unidentified mechanism.

Primary treatment of DADA2 relies on anti-TNF agents in patients with a mostly inflammatory phenotype, while patients with bone marrow failure may require HSCT (124, 125). Identification of patients with ADA1-SCID and DADA2 has helped identify unexpected roles for adenosine as a key molecule in the regulation of immune physiology.

### Interferonopathies

Interferonopathies are a group of systemic inflammatory diseases with continuum of features of autoinflammation and autoimmunity. The underlying mechanism of inflammation in monogenic interferonopathies is a defect in sensing and degradation of nucleic acids leading to constitutive activation of innate immune responses (126, 127). These disorders show a striking resemblance to congenital viral infections although occur in the absence of any identified pathogen.

Two of the best studied diseases are Aicardi-Goutières syndrome (AGS) and stimulator of interferon genes (STING)-associated vasculopathy with onset in infancy (SAVI). AGS is a heterogenous early-onset disorder manifesting with basal ganglia calcifications, encephalopathy, neurological impairments and other features of autoimmunity. Disease-associated mutations have been identified in genes that either impact nucleic acid degradation (*TREX1, SAMHD1, RNASEH2A, RNASEH2B, RNASEH2C, DNASE2)*, or sensing of nucleic acids (*IFIH1/*MDA5) or RNA editing *(ADAR)* and result in activation of the type I interferon pathway. Patients with SAVI have gain-of-function mutations in *TMEM173*, which encodes the STING protein, and they present with prominent skin lesions, small vessel vasculitis, peripheral amputations, and interstitial lung disease. STING is an endoplasmic transmembrane protein that functions as an indirect sensor of endogenous or pathogen-derived cytosolic dsDNA (128). Upon binding to cGAMP, which is generated by the sensing receptor cGAS, STING activates IRF3, a transcription factor for type 1 interferon and related genes. Production of type 1 interferon cytokines results in an amplification loop that can be blocked by JAK inhibitors providing further insights into the mechanism of pathogenesis (129). Type I interferonopathies mechanisms, disease manifestations, and treatment have been reviewed in great detail elsewhere (127, 130).

## Conclusions

**Figure 6:**
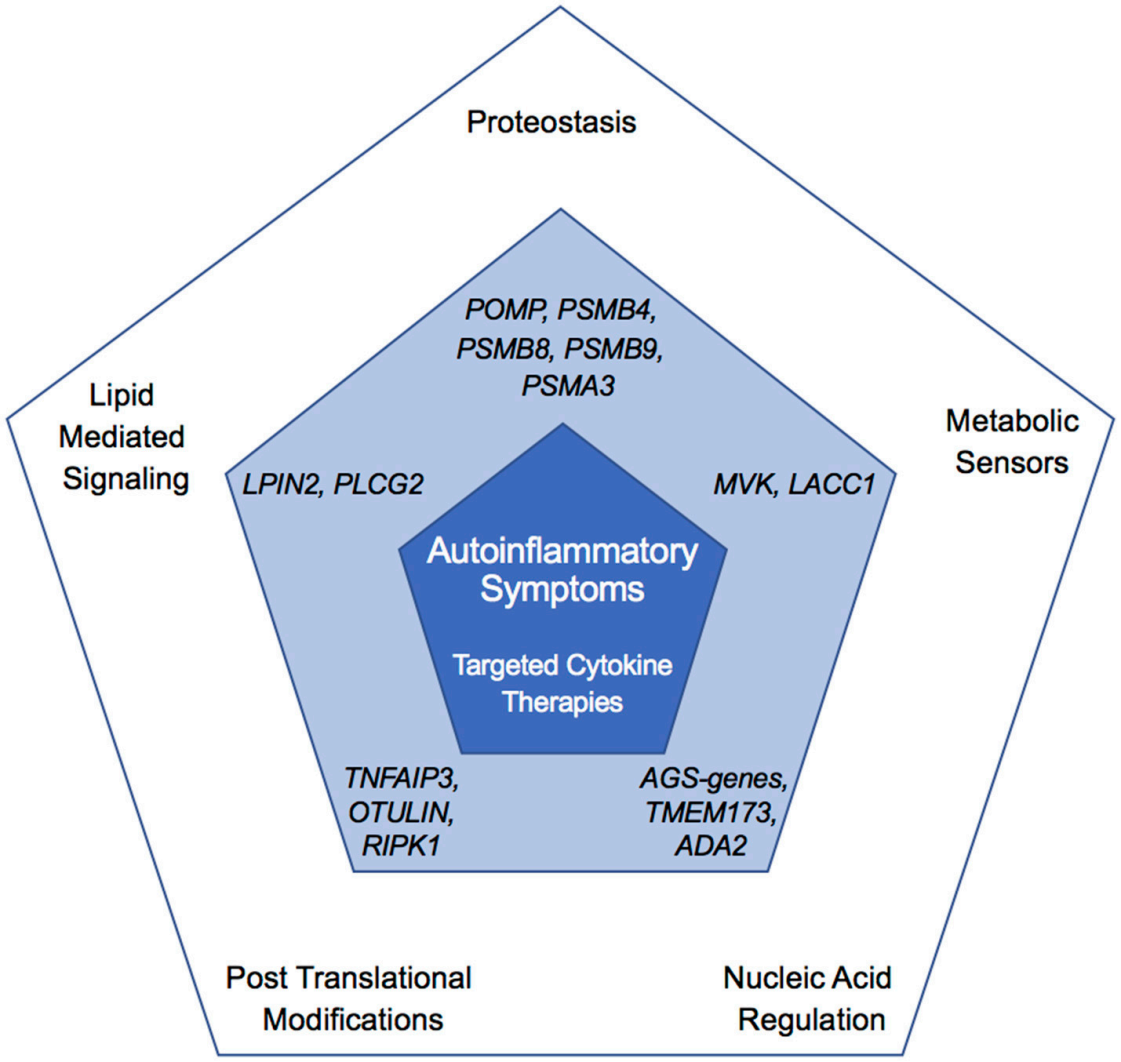
Overlapping and unique features of autoinflammatory diseases. Outer pentagon shows pathways altered in autoinflammatory disease, central pentagon highlights genes in the corresponding node found mutated in monogenic disease (dark blue), and inner pentagon showing shared clinical features and treatment (light blue).

The disorders discussed in this review exemplify how unbiased identification of mutated genes in patients with rare autoinflammatory diseases has uncovered novel mechanisms governing innate immune responses. Collectively, these findings point to previously unrecognized functions for these genes and their associated pathways. These atypical autoinflammatory genes not only help identify unique pathways regulating inflammatory pathways, but also illuminate shared features with other disorders based on treatment response and clinical manifestations (Figure 6). It is unclear whether these genes have a specific function in immune system regulation or if the relatively specific immune phenotypes are due to particular sensitivity of the immune system to disruption in basic cell machinery and metabolism. Furthermore, unlike disruption of canonical autoinflammatory pathways, mutations in many of these genes can result in concomitant overactivity of the innate immune system (autoinflammation) and of the adaptive immune system (autoimmunity) and be accompanied with ineffective immune responses (immunodeficiency). This provides evidence for cell-specific function of these enzymes in the regulation of metabolic and immune signaling pathways. Besides targeted anti-cytokine therapies, hematopoietic stem cell transplantation has been attempted in some patients refractory to conventional treatments, and can cure hematological and immunological manifestations. Like with other metabolic disorders, early diagnosis and treatment is critical for preventing disease complications. Unbiased genetic sequencing of patients with early-onset immune dysregulation disorders will continue to identify novel disease-causing genes and further delineate the molecular mechanisms governing inflammation in humans.

## Web Link

https://infevers.umai-montpellier.fr/web.

## Author Contributions

All authors listed have made a substantial, direct and intellectual contribution to the work, and approved it for publication.

### Conflict of Interest Statement

The authors declare that the research was conducted in the absence of any commercial or financial relationships that could be construed as a potential conflict of interest.
